# The Expanding Role of ChatGPT (Chat-Generative Pre-Trained Transformer) in Neurosurgery: A Systematic Review of Literature and Conceptual Framework

**DOI:** 10.7759/cureus.43502

**Published:** 2023-08-15

**Authors:** Alex Roman, Lubna Al-Sharif, Mohamed AL Gharyani

**Affiliations:** 1 Neurological Surgery, Cleveland Clinic Abu Dhabi, Abu Dhabi, ARE; 2 Physiology, Pharmacology and Toxicology, An-Najah National University, Nablus, PSE; 3 Neurosurgery, University of Benghazi, Benghazi, LBY

**Keywords:** neurosurgery, deep learning, chatgpt, artificial intelligence, ai & robotics in healthcare

## Abstract

The objective of this study is to explore the use of ChatGPT (Chat-Generative Pre-Trained Transformer) in neurosurgery and its potential impact on the field. The authors aim to discuss, through a systematic review of current literature, how this rising new artificial intelligence (AI) technology may prove to be a useful tool in the future, weighing its potential benefits and limitations. The authors conducted a comprehensive and systematic literature review of the use of ChatGPT and its applications in healthcare and different neurosurgery topics. Through a systematic review of the literature, with a search strategy using the databases such as PubMed, Google Scholar, and Embase, we analyzed the advantages and limitations of using ChatGPT in neurosurgery and evaluated its potential impact. ChatGPT has demonstrated promising results in various applications, such as natural language processing, language translation, and text summarization. In neurosurgery, ChatGPT can assist in different areas such as surgical planning, image recognition, medical diagnosis, patient care, and scientific production. A total of 128 articles were retrieved from databases, where the final 22 articles were included for thorough analysis. The studies reviewed demonstrate the potential of AI and deep learning (DL), through language models such as ChatGPT, to improve the accuracy and efficiency of neurosurgical procedures, as well as diagnosis, treatment, and patient outcomes across various medical specialties, including neurosurgery. There are, however, limitations to its use, including the need for large datasets and the potential for errors in the output, which most authors concur will need human verification for the final application. Our search demonstrated the potential that ChatGPT holds for the present and future, in accordance with the studies’ authors’ findings herein analyzed and expert opinions. Further research and development are required to fully understand its capabilities and limitations. AI technology can serve as a useful tool to augment human intelligence; however, it is essential to use it in a responsible and ethical manner.

## Introduction and background

Artificial intelligence (AI) has shown significant potential in enhancing medical care, with immense possibilities in the field of neurosurgery as neural processing systems rapidly advance [1]. Among the emerging AI technologies, ChatGPT (Chat-Generative Pre-Trained Transformer) stands out as a promising language model developed and uniquely trained by artificial intelligence laboratory research. ChatGPT has demonstrated promising results in various applications [2], including language translation, text summarization, and natural language processing. Its potential for medical documentation, healthcare data processing, diagnosis, research, and education is particularly noteworthy [2]. Exploring the use of ChatGPT in neurosurgery and understanding its potential as a valuable tool in the future is of great interest.

The field of neurosurgery has witnessed significant advancements, driven mainly by rapidly evolving technologies that have revolutionized patient care and improved outcomes over the past few decades. The emergence of ChatGPT stems from the integration of artificial intelligence (AI) and natural language processing (NLP). This novel technology tool has gained recognition for its potential applications, particularly in healthcare fields [2]. ChatGPT, as an AI language model, utilizes deep learning to generate human-like responses to natural language queries. Its potential to enhance medical practice, such as improving surgical planning, analyzing imaging data, and manuscript writing across various medical disciplines, including neurosurgery, is noteworthy but has yet to be heedfully analyzed [3-5].

In neurosurgical practice, ChatGPT can serve as an intelligent assistant, aiding clinicians in making informed decisions based on patient data. By analyzing patient information, including imaging studies and laboratory results, ChatGPT can provide recommendations on diagnosis, treatment, and prognosis. However, it is essential to verify its competency in delivering reliable and trustworthy information [1]. Additionally, ChatGPT can streamline communication between clinicians and patients, enabling real-time interpretation of patient needs and enhancing the quality of care provided.

The utilization of ChatGPT in imaging analysis is gaining prominence. It has the potential to assist in the interpretation of complex imaging studies, helping identify potential abnormalities that may be overlooked by human interpretation alone. However, preprocessing of imaging data would be necessary before the final application. This has the potential to improve the accuracy of diagnosis and treatment planning, ultimately leading to better patient outcomes.

Beyond medical practice and imaging analysis, ChatGPT can also be harnessed for scientific literature production [5]. Manuscript writing is a labor-intensive process that demands extensive research and revision. By generating outlines, identifying relevant literature, and offering suggestions for sentence structure and grammar, ChatGPT can streamline the writing process for researchers. This could significantly reduce the time and effort required to produce high-quality scientific manuscripts, although questions may arise regarding the potential loss of individual creativity [6-10]. This article aims to discuss, therefore, the potential applications of ChatGPT in different neurosurgery topics and its impact on the field.

## Review

Methods

In order to investigate the utilization of ChatGPT in neurosurgery, we conducted a comprehensive and systematic literature review, focusing on the application of AI in healthcare, specifically within the neurosurgery specialty. Our search encompassed peer-reviewed journals, including primary research studies and review articles. To ensure a comprehensive understanding of the current state of the field, we also considered gray literature, such as conference proceedings and preprints. We analyzed the advantages and limitations of implementing ChatGPT in neurosurgery and evaluated its potential impact on the field. The study was designed following the guidelines of the Preferred Reporting Items for Systematic Reviews and Meta-Analyses (PRISMA). Prior to finalizing and approving the study's conceptual construction, it was registered in the international systematic review database, International Prospective Register of Systematic Reviews (PROSPERO), with the protocol registry number CRD42023434391 (http://www.crd.york.ac.uk/prospero/).

**Figure 1:**
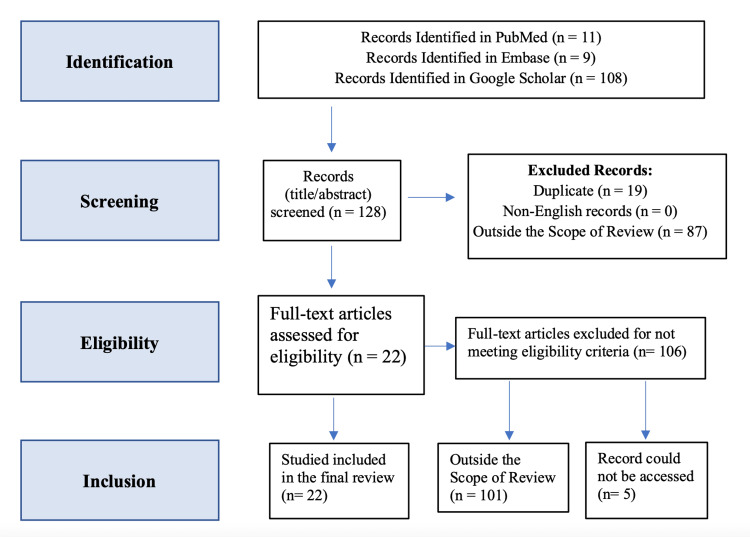
Flowchart of the articles selection process based on the Preferred Reporting Items for Systematic Review and Meta-Analysis (PRISMA) guidelines

A thorough review of the literature was conducted using multiple electronic databases, including PubMed, Embase, and Google Scholar. The search terms employed were "neurosurgery" AND "ChatGPT", utilizing the advanced search tool of PubMed with the following terms: "chatgpt"[All Fields] AND ("neurosurgery"[MeSH Terms] OR "neurosurgery"[All Fields] OR "neurosurgeries"[All Fields] OR "neurosurgery s"[All Fields] OR "neurosurgical procedures"[MeSH Terms] OR ("neurosurgical"[All Fields] AND "procedures"[All Fields]) OR "neurosurgical procedures"[All Fields]). The inclusion criteria for the articles were as follows: being written in English, published within the last 10 years, and describing the potential benefits and limitations of AI and language processing software, specifically ChatGPT, in the context of neurosurgery (Figure 1). Studies that did not pertain to the use of ChatGPT and AI in medicine or those that were not focused on ChatGPT in healthcare were excluded. The search concluded on June 30, 2023. Two independent researchers worked separately and collectively determined the endpoint of the search to ensure consistent results. In cases of disagreement, a third researcher served as the deciding vote.

Following the screening of titles and abstracts of the retrieved articles, full-text versions of the relevant studies were obtained and thoroughly reviewed. Additionally, the references of the selected studies were examined to identify any additional pertinent articles that were initially not retrieved. To ensure originality and avoid plagiarism, all the text in this study was written by the authors, and any quoted or paraphrased information was properly cited. Furthermore, plagiarism detection software, Turnitin, was employed to check the entire text for unintentional similarity with existing works. To mitigate biases and conflicts of interest, we ensured that the authors had no financial or other conflicts that could have influenced the study's outcomes.

Results

The comprehensive literature review yielded 22 articles that explored the application of Chat Generative Pre-Trained Transformer (ChatGPT) in healthcare and neurosurgery, shedding light on its potential benefits and limitations (Table 1) [2,5-7,10-31]. Some articles highlighted ChatGPT's capability to support medical professionals by providing accurate information and answering queries based on patient data analysis, aiding, therefore, in decision-making processes [2,11]. However, concerns were also raised about potential misinformation and the need for human oversight to ensure patient safety and address ethical considerations [2,11].

**Table 1:** ChatGPT in Healthcare and Neurosurgery

	Article Title	First Author, Year	Study Design	Conclusions/Recommendations
1.	Benefits, limits, and risks of GPT-4 as an AI chatbot for medicine	Lee et al. [1], 2023	Literature review and analysis	GPT-4 has the potential to assist in medical education and decision-making, but careful consideration of ethical and legal issues is necessary.
2.	AI-generated research paper fabrication and plagiarism in the scientific community	Elali and Rachid [2], 2023	Literature review and analysis	AI-generated papers can be used for plagiarism, highlighting the need for academic integrity policies and software.
3.	Cyberknife radiosurgery for synovial sarcoma metastasizing to the spine: illustrative case reports	Zamarud et al. [3], 2023	Case reports	Cyberknife radiosurgery can be an effective treatment for metastasizing synovial sarcoma.
4.	Internal carotid artery pseudoaneurysm after transsphenoidal pituitary tumor resection: a case report	Montagne et al. [4], 2023	Case report	Careful surgical techniques can help prevent complications such as internal carotid artery pseudoaneurysm.
5.	The rise of artificial intelligence: addressing the impact of large language models such as ChatGPT on scientific publications	Ang et al. [5], 2023	Literature review and analysis	ChatGPT and other large language models can revolutionize scientific publication, but ethical and legal concerns must be addressed.
6.	Currarino syndrome presenting in adulthood: a rare case	Verma et al. [17], 2023	Case report	Currarino syndrome can present in adulthood and requires appropriate diagnostic and management strategies
7.	Long-term survival of patients with glioblastoma of the pineal gland: a ChatGPT-assisted, updated case of a multimodal treatment strategy resulting in extremely long overall survival at a site with historically poor outcomes	Cunningham et al. [11], 2023	Case report	ChatGPT can assist in the management of glioblastoma of the pineal gland, leading to prolonged overall survival.
8.	ChatGPT in education: strategies for responsible implementation	Halaweh [6], 2023	Literature review and analysis	ChatGPT can enhance education but requires responsible implementation and monitoring.
9.	Treatment outcomes of leiomyosarcoma metastasis affecting the brachial plexus: a comparative case report using Chat Generative Pre-Trained Transformer (ChatGPT)	Zamarud et al. [8], 2023	Case report	ChatGPT can assist in treatment planning for leiomyosarcoma metastasis affecting the brachial plexus.
10.	Utility in health care education, research, and practice: systematic review on the promising perspectives and valid concerns	Sallam [7], 2023	Systematic review	ChatGPT has potential in various areas of healthcare, but further research and development are needed to address concerns such as privacy, bias, and accuracy.
11.	neuroGPT-X: towards an accountable expert opinion tool for vestibular schwannoma	Guo et al. [9], 2023	Development of neuroGPT-X	neuroGPT-X has the potential to assist with clinical decision-making in vestibular schwannoma cases, but further research and validation are needed.
12.	Extraventricular neurocytoma of the posterior fossa: a case report written by ChatGPT	Hegde et al. [24], 2023	Case report	ChatGPT can generate accurate and detailed case reports for medical purposes.
13.	ChatGPT in glioma patient adjuvant therapy decision making: ready to assume the role of a doctor in the tumor board?	Haemmerli et al. [12], 2023	Retrospective study	ChatGPT shows promise in assisting with adjuvant therapy decision-making for glioma patients but requires further validation and integration into clinical practice.
14.	On chatbots and generative artificial intelligence	Oermann and Kondziolka [13], 2022	Review article	Chatbots have potential in healthcare, but ethical and legal considerations must be addressed, and further research is needed to determine their effectiveness and limitations.
15.	Performance of ChatGPT and GPT-4 on neurosurgery written board examinations	Ali et al. [14], 2023	Comparative study	ChatGPT and GPT-4 show potential in assisting with neurosurgery board exam preparation, but further research and development are needed.
16.	ChatGPT versus the neurosurgical written boards: a comparative analysis of artificial intelligence/machine learning performance on neurosurgical board-style questions	Hopkins et al. [15], 2023	Comparative study	ChatGPT shows promise in assisting with neurosurgery board exam preparation but requires further development to address limitations in accuracy and relevance.
17.	I asked a ChatGPT to write an editorial about how we can incorporate chatbots into neurosurgical research and patient care…	D'Amico et al. [23], 2023	Qualitative analysis of ChatGPT generated editorial	Chatbots can provide support for neurosurgical research and patient care through providing easy access to medical information, assistance with patient education, and improving communication between healthcare professionals and patients.
18.	The role of an open artificial intelligence platform in modern neurosurgical education: a preliminary study	Sevgi et al. [10], 2023	Analysis of an AI education platform with student feedback	An open artificial intelligence platform can provide valuable educational resources for neurosurgical trainees, improve learning outcomes, and promote collaborative learning.
19.	The potential impact of ChatGPT/GPT-4 on surgery: will it topple the profession of surgeons?	Cheng et al. [19], 2023	Literature review and discussion	Chatbots and AI may have the potential to augment the role of surgeons, improve patient outcomes, and increase efficiency in surgical care. However, it is unlikely that AI will completely replace the profession of surgeons.
20	Emergency surgery in the era of artificial intelligence: ChatGPT could be the doctor's right-hand man	Cheng et al. [20], 2023	Discussion and analysis	Chatbots such as ChatGPT can play a valuable role in emergency surgery by assisting with preoperative evaluation, providing decision support, and facilitating communication between healthcare professionals.
21.	GPT-4: a new era of artificial intelligence in medicine	Waisberg et al. [16], 2023	Literature review and discussion	GPT-4 has the potential to revolutionize medicine by enhancing clinical decision-making, improving communication with patients, and advancing medical research. However, there are still ethical and legal concerns to address.
22.	Implications and future directions of ChatGPT utilization in neurosurgery	Singh et al. [26], 2023	Review article	ChatGPT has potential in neurosurgery, including in diagnostics, data analysis and clinical decision, education and administrative assistance. Albeit, inaccuracies in the output of information and medicolegal consequences must be emphasized.

In the realm of scientific publications, researchers addressed AI-generated research paper fabrication and plagiarism, emphasizing the importance of detecting and preventing AI-generated plagiarism to uphold the integrity of scholarly works [2]. Moreover, ChatGPT was showcased as a valuable tool for enhancing medical case reporting and study data analysis for manuscript production [8,9,11,12]. The impact of ChatGPT on medical education was explored, with discussions centering on strategies for responsible implementation to enhance learning experiences, promote critical thinking, and address individual learning needs in healthcare education [10].

ChatGPT's potential in specialized medical areas was showcased through its role in assisting in establishing prognosis and supporting adjuvant therapy decisions in glioma patients, with its potential for oncology application [24-28]. Furthermore, the tool was explored in practical neurosurgery for research and patient care, raising ethical questions and discussing its limitations while emphasizing the importance of human verification [22,23]. Various case reports demonstrated ChatGPT's role in analyzing and reporting on specific medical cases, such as papers by Zamarud et al. [3] and Montagne et al. [4]. The tool showcased its potential in enhancing medical case reporting and study data analysis.

Overall, the reviewed articles underscore the potential of AI and deep learning, including ChatGPT, to enhance the accuracy and efficiency of neurosurgical procedures, utilizing surgical planning, diagnosis, further treatment options, and patient outcomes across various settings of neurosurgery [1,11,16,18,24-28]. However, the importance of developing transparent and interpretable AI models is emphasized, alongside the need for further research to validate the clinical utility of AI applications in neurosurgery and address ethical, legal, and social implications [1,5,18]. While ChatGPT holds promise for future research in neurosurgery, careful consideration of its limitations and ethical implications remains essential [26-28]. The potential of AI and deep learning to revolutionize healthcare, including the neurosurgery field, is evident, but it is crucial to approach its implementation responsibly to ensure patient safety and promote ethical use.

Discussion

ChatGPT, a machine learning-powered language model, exhibits tremendous promise across various aspects of neurosurgery, although limitations need to be considered [1]. For example, in surgical planning, ChatGPT swiftly generates reports and analyzes medical records, proving to be a valuable resource for neurosurgeons. By capturing patterns that might elude human physicians, it assists in assessing the risks and benefits of different surgical approaches, ultimately guiding the determination of the most suitable treatment plan for individual patients [29]. Additionally, artificial intelligence, including ChatGPT, proves invaluable in handling vast patient data, providing valuable insights into surgical prognosis, overall survival, and morbidity prediction [30].

ChatGPT's proficiency in image analysis shines through its accurate recognition of anatomical structures and abnormalities in various medical images, such as MRI scans, CT scans, and X-rays. This enhanced diagnostic accuracy allows clinicians to identify potential complications and postoperative outcomes based on a patient's medical history. Furthermore, the technology finds applications in oncology diagnosis and treatment planning, further solidifying its significance in the field [31]. However, careful validation and the inclusion of human verification remain imperative, particularly in critical medical decision-making scenarios.

Moreover, medical diagnosis benefits from ChatGPT's ability to generate differential diagnoses based on patient symptoms and medical history. Its capacity to analyze extensive medical data contributes to precise diagnoses and well-informed treatment plans in numerous cases. Nonetheless, a balanced approach requires acknowledging its limitations and, again, the necessity for careful validation. Transfer learning emerges as an effective approach to enhance the performance of deep learning models, as demonstrated in brain lesion segmentation [32]. Beyond diagnosis and planning, ChatGPT proves to be a valuable resource in delivering personalized medical advice and education to patients. By providing information on treatment options, potential risks, and benefits, as well as postoperative care, it enhances patient satisfaction and engagement [25]. Notwithstanding these applications, furthermore, ChatGPT has been shown to aid in scientific production, facilitating text generation, with a number of scientific articles being produced by such means, as well as being an adjuvant in education [8-12].

However, like any technological advancement, ChatGPT presents certain limitations in neurosurgery. Notably, a significant challenge lies in the demand for extensive datasets, which may pose obstacles due to patient confidentiality and privacy concerns. Additionally, potential errors in output necessitate meticulous validation to ensure accuracy and reliability. Being mindful of these limitations while capitalizing on its benefits is essential to the responsible integration of AI in neurosurgery [1,26]. While our study has been a comprehensive and systematic review, it is possible that the rapidly evolving research landscape on ChatGPT and other novel AI tools in healthcare may lead to inadvertent exclusions of important studies. Furthermore, studies not translated into English might have been unintentionally omitted, underscoring the need for ongoing vigilance to capture significant developments in the field.

In summary, ChatGPT offers exciting prospects in neurosurgery, demonstrating its potential as a powerful tool for surgical planning, image analysis, medical diagnosis, and patient care. The benefits it brings are noteworthy, but careful consideration of its limitations and validation is crucial. By adopting a responsible approach to leverage artificial intelligence, the medical field can fully harness ChatGPT's potential, ultimately enhancing patient outcomes and advancing the practice of neurosurgery. Looking ahead, collaboration between neurosurgeons, healthcare professionals, AI experts, and data scientists is crucial to integrating AI into clinical practice in a transparent and effective manner. By working together, we can maximize the benefits of ChatGPT and AI while mitigating potential risks and ensuring the highest standards of patient care.

## Conclusions

ChatGPT presents a promising AI technology that holds the potential to revolutionize neurosurgery in the future. Its promising applications in various medical modalities, including surgical planning, image recognition, medical diagnosis, and patient care, have shown significant potential, as demonstrated in this study's analysis of current literature. By assisting in decision-making and reducing the workload of neurosurgeons, ChatGPT offers the opportunity for more creativity within the specialty. However, it is essential to acknowledge the limitations of using ChatGPT in neurosurgery, such as the requirement for large datasets and the possibility of errors in output. Further research and development are necessary to fully comprehend its capabilities and limitations.
